# Successful selection of mouse sperm with high viability and fertility using microfluidics chip cell sorter

**DOI:** 10.1038/s41598-020-65931-z

**Published:** 2020-06-01

**Authors:** Satohiro Nakao, Toru Takeo, Hitomi Watanabe, Gen Kondoh, Naomi Nakagata

**Affiliations:** 1 Division of Reproductive Engineering, Center for Animal Resources and Development, Kumamoto University, 2-2-1 Honjo, Chuo-ku, Kumamoto 860-0811 Japan; 2 Laboratory of Integrative Biological Science, Institute for Frontier Life and Medical Sciences, Kyoto University, 53 Shogoinkawahara-cho, Sakyo-ku, Kyoto 606-8507 Japan

**Keywords:** Biotechnology, Reproductive biology

## Abstract

Cell sorting via flow cytometry is a powerful tool to select subpopulations of cells in many biological fields. Selection of fertilisation-prone sperm is a critical step to ensure a stable and high fertilisation rate in *in vitro* fertilisation (IVF). However, a combination of conventional cell sorting and IVF system has not been established because of severe mechanical damages to the sperm during the sorting process. A cell sorter with microfluidics chip technology that lessens cell damage during cell sorting may address this problem. We evaluated the effects of microfluidics chip cell sorting on the sperm using the parameters, such as motility and fertility, and found this cell sorting method had minimal harmful effect on the sperm. Then, sperm were selected by a marker for acrosome reaction and showed higher fertilisation rate than that of the population of acrosome-intact sperm. Embryo derived from these sperm developed normally. These results indicated that microfluidics chip cell sorting can select fertile sperm to improve IVF technique.

## Introduction

Selection of fertile sperm is a key step in obtaining a stable and high fertilisation rate in *in vitro* fertilisation (IVF). Sperm selection is generally performed according to motility, morphological integrity and specific gravity, using the swim-up method and density gradient centrifugation^1–4^. Therefore, with the aim of improving the IVF technology, we wanted to employ other methods such as sperm sorting as well as conventional methods.

Cell sorting using flow cytometry is a powerful method for selecting subpopulation of cells in many biological studies involving various indicators of fluorescence proteins, antibodies and compounds. However, conventional cell sorting is not suitable for fragile cells such as sperm of various mammalian species.

Recently, a microfluidics chip cell sorter has been developed to cope with vulnerable cells, including cancer, blood and immune cells^5–9^. With regards to the core techniques of cell sorting, conventional cell sorters use electric charge to separate cells, whereas the microfluidics chip cell sorter separates cells using pulsed air pressure, which results in minimal damages to cells^10^. Therefore, we considered that the microfluidics chip cell sorter may be useful to select fertile sperm.

In this study, we first examined whether the microfluidics chip cell sorter is useful for sperm selection by using the parameters such as motility, fertility and developmental ability of the collected sperm. Then, we selected acrosome-reacted sperm using fluorescein isothiocyanate (FITC, a fluorescence compound)-labelled peanut agglutinin (PNA) to demonstrate the efficacy of this system.

## Results

### Motility parameters of sperm sorted using the microfluidics chip cell sorter

**Figure 1:**
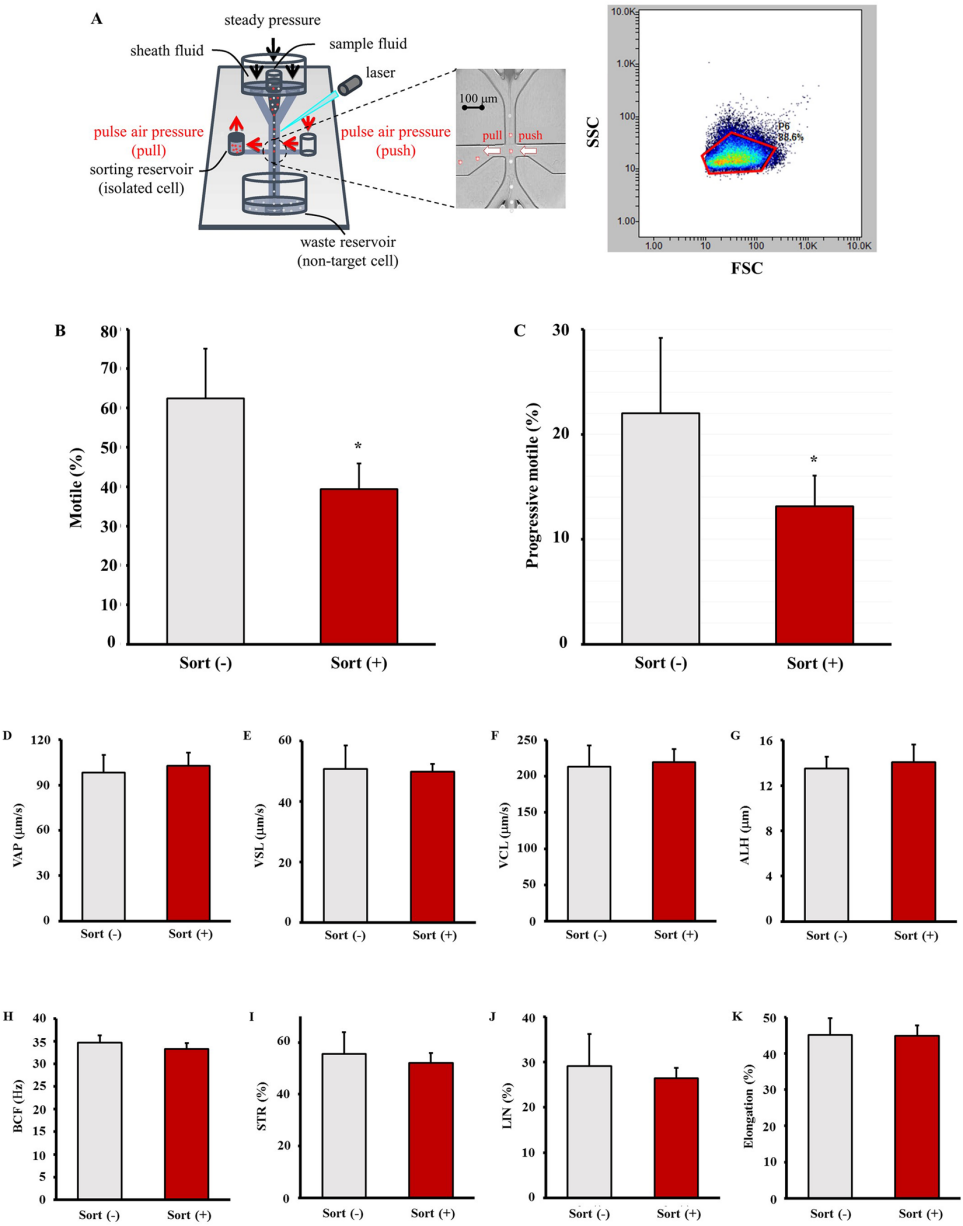
Effect of sorting on sperm motility. Epididymal sperm were sorted using a signal distribution of forward scattered light (FSC) and side scattered light (SSC) (**A**), and sperm motility was analysed using a HTM-IVOS. Motility is the ratio of sperm moving 5 µm/s to total sperm (**B**). Progressive motility is the ratio of sperm moving at 50 µm/s to total sperm and with straightness greater than 50% (**C**). Path velocity (VAP) is average velocity of the smoothed sperm path (**D**). Progressive velocity (VSL) is the average velocity measured in a straight line from the beginning to the end of the sperm track (**E**). Track speed (VCL) is the average velocity measured over the actual point to point sperm track (**F**). Lateral amplitude (ALH) is the mean width of the head oscillation as the sperm swims (**G**). Beat frequency (BCF) is the frequency of sperm heads crossing the average sperm path in either direction (**H**). Straightness (STR) is a measure of the departure of the average sperm path from a straight line (ratio of VSL/VAP) (**I**). Linearity (LIN) is a measure of the departure of the actual sperm track from a straight line (ratio of VSL/VCL) (**J**). Elongation is the ratio of head width to head length (**K**). Values are given as the mean ± SD (n = 7). *p < 0.05 compared with sort (−). We acknowledged that the copyright of Fig. 5A was held by On-chip Biotechnologies.

Initially, we tried to collect motile sperm using a conventional flow cytometer; however, we could only obtain a small number of sperm whose tails were moving up and down (Supplemental Video 1). Alternatively, we used the microfluidics chip cell sorter to collect intact sperm as much as possible. In addition, the sample after sorting was collected in almost un-diluted state ready to use for IVF. Sperm were sorted by the microfluidics chip cell sorter based on the signal distribution of forward scattered light (FSC) and side scattered light (SSC) (Fig. 1A). As shown in Supplemental Video 2, we could obtain sufficient number of motile sperm after sorting.

To examine the viability of the sorted sperm, the motility of sorted and unsorted sperm was compared. Although motile sperm were obtained after the cell sorting, the percentages of motile sperm and progressive motile sperm in the sorted sperm were lower than in the unsorted sperm (Figs. 1B,C). Conversely, other parameters of the sorted sperm were equivalent to those of the unsorted sperm (Fig. 1D–K).

### Fertility and developmental abilities of the sorted sperm

**Figure 2:**
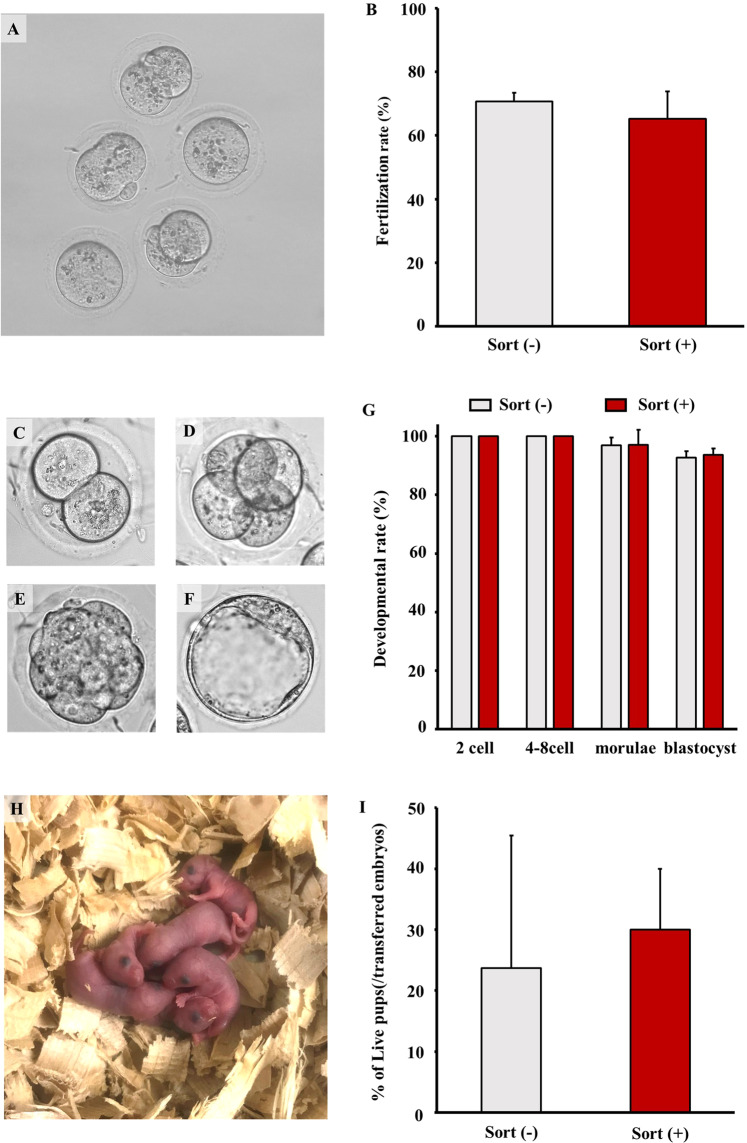
Effect of sorting on sperm fertility and developmental ability. Sperm were pre-incubated for 60 min, and sperm suspensions were diluted using mHTF to adjust the flow rate to approximately 400 events/s. Then, cell sorting was performed using forward scattered light (FSC) and side scattered light (SSC). After sorting, the sorted sperm suspensions were collected from the sorting reservoir and used as *in vitro* fertilisation (IVF) medium. Oocytes were introduced into the IVF medium. After IVF, the two-cell embryos were either cultured in KSOM/AA medium or transferred into the oviducts of pseudopregnant ICR females (10 embryos/oviducts). (**B**) The fertilisation rate was calculated as the number of two-cell embryos (**A**) divided by the number of inseminated oocytes x 100. (**G**) The developmental rate was calculated as the number of embryos at several stages— 4-cell (**D**), morulae (**E**) and blastcysts (**F**)—divided by the number of two-cell embryos (**C**) x 100. (**H**) Two-cell embryos developed normally into live pups. I) the birth rate was calculated as the (number of live pups divided by the number of transferred two-cell embryos) x 100. Values are given as the mean ± SD (n = 3-6). *p < 0.05 compared with sort (−).

To examine the fertility of the sorted sperm, they were used in IVF, and it was found that in comparison to unsorted sperm, sorted sperm fertilised with oocytes and fertilised eggs developed normally up to the blastocyst stage in *in vitro* culture (Fig. 2A–G). When the embryos were transferred to the pseudopregnant females, we obtained normal pups with birth rate similar to those of the unsorted sperm (Fig. 2G–I). These observations suggest that sperm sorted by the microfluidics chip cell sorter have normal fertilisation and developmental ability.

### Selection of acrosome-reacted sperm by cell sorting

**Figure 3:**
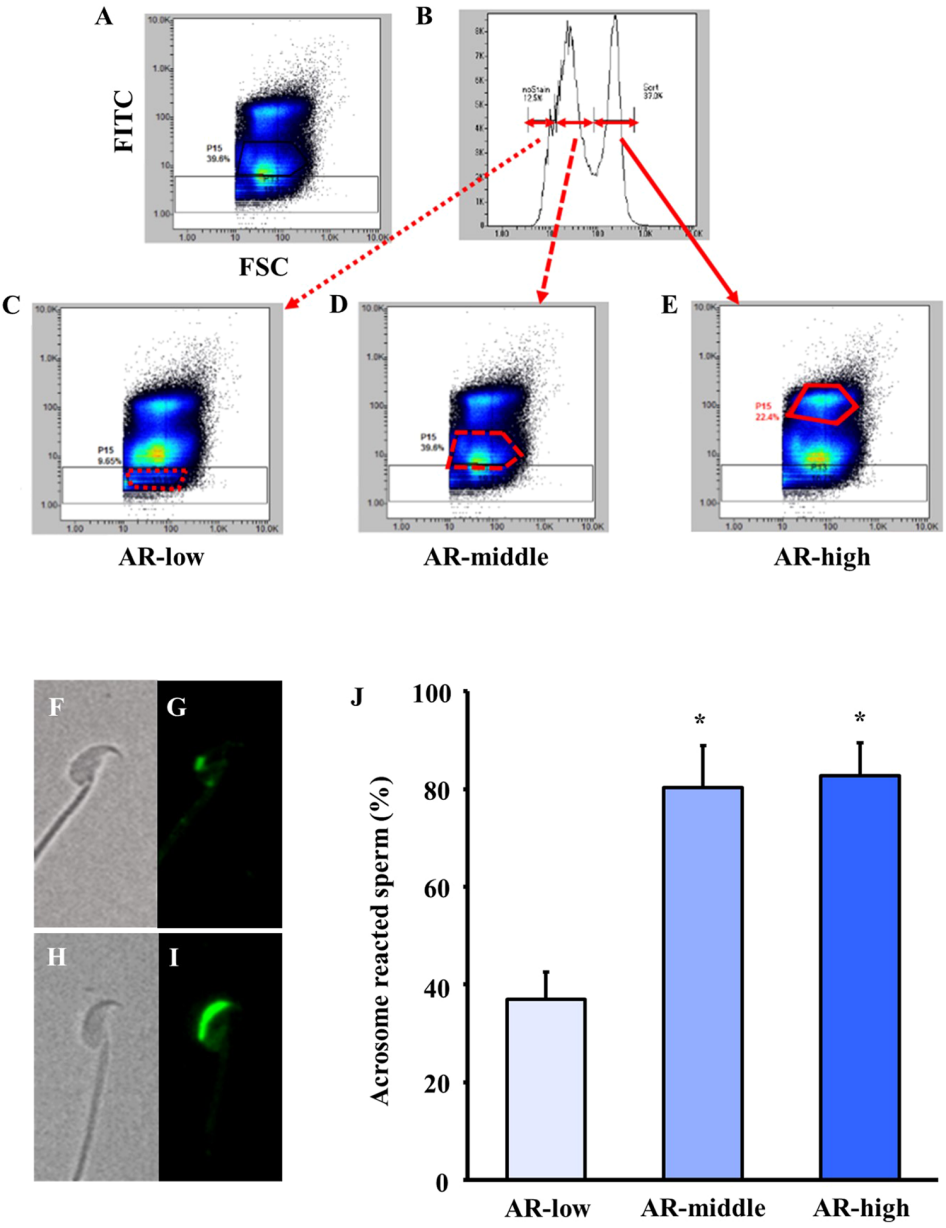
Rate of acrosome-reacted sperm after selection. Sperm samples were dissolved in mHTF, and the flow rate was adjusted to approximately 400 events/s. The sperm were gated into fluorescence intensity of FITC-labelled PNA and collected in the collection reservoir. (**A–E**) We classified the sperm into three groups— AR-low, AR-middle and AR-high—according to intensity of FITC. After selection, the sperm were observed under fluorescence microscopy, and the number of sperm stained green were counted as acrosome-reacted sperm, AR (+). F, G) Fluorescence image of acrosome-intact sperm, AR ( − ). H, I) Fluorescence image of AR (+). J) The rate of acrosome-reacted sperm (%) was calculated as the number of AR (+) divided by the total number of AR (+) and AR (−). Values are given as the mean ± SD (n = 3). *p < 0.05 compared with AR-low.

To examine the effect of sperm selection based on acrosome reaction (AR) on fertilisation ability, sperm were stained with FITC-labelled PNA and sorted based on the signal distribution of forward scattered light (FSC) and signal intensity of FITC (acrosome-intact, FITC negative: acrosome-reacted, FITC positive). The sperm were classified into three groups based on the signal intensity of FITC [acrosome-reacted low (AR-low), acrosome-reacted middle (AR-middle) and acrosome-reacted high (AR-high)] and collected separately (Fig. 3C–E). Observation under fluorescence microscopy revealed that more than 80% of the acrosome-reacted sperm were in the AR-middle and AR-high groups (Fig. 3J). Conversely, in the AR-low group, the population of acrosome-reacted sperm was reduced by almost half. Therefore, acrosome-reacted sperm were enriched by the microfluidics chip cell sorter.

**Figure 4:**
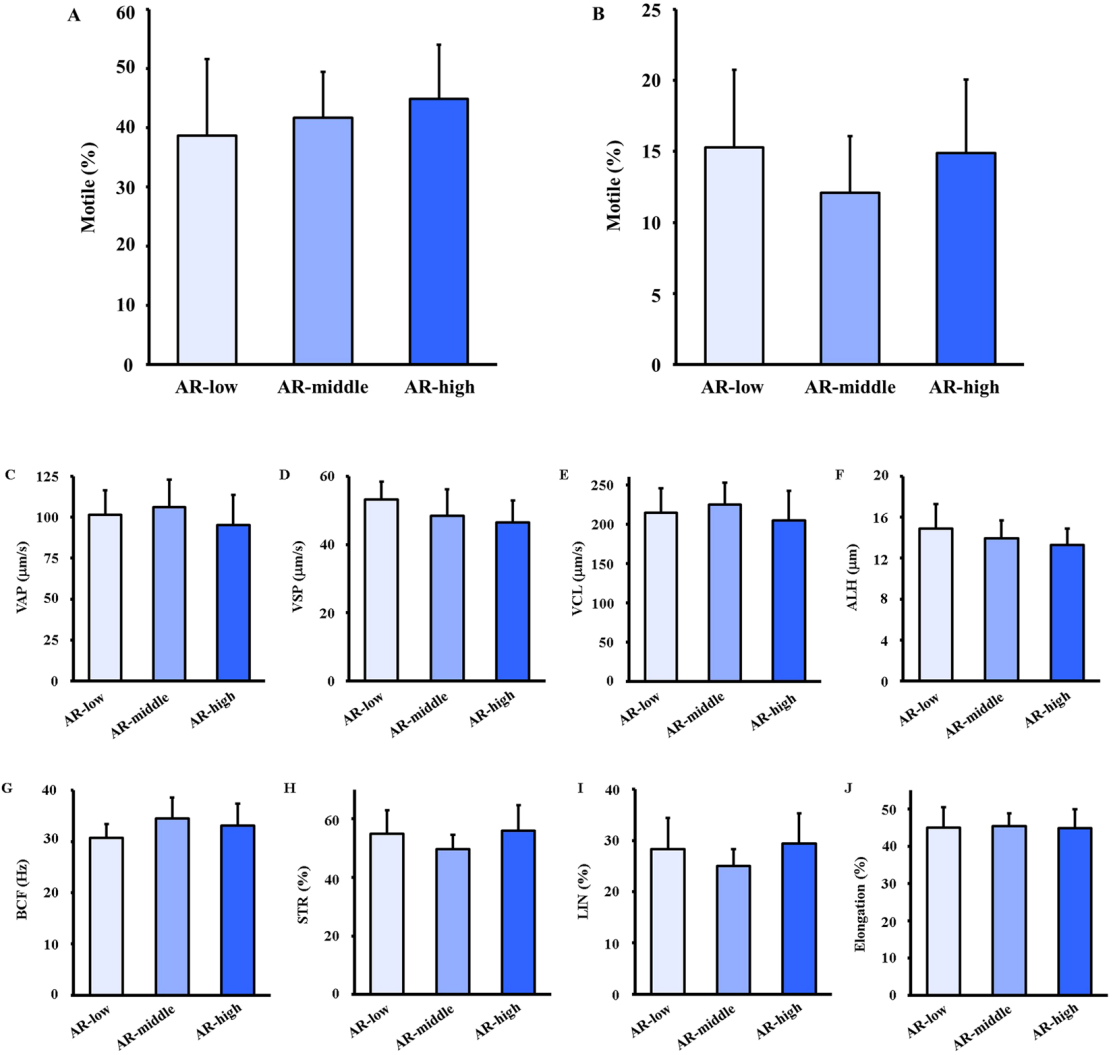
Effect of selection on sperm motility. After selection, sperm motility was analysed using an HTM-IVOS. Motility is the ratio of sperm moving 5 µm/s to total sperm (**A**). Progressive motile is the ratio of sperm moving at 50 µm/s to total sperm and swith traightness higher than 50% (**B**). Path velocity (VAP) is the average velocity of the smoothed sperm path (**C**). Progressive velocity (VSL) is the average velocity measured in a straight line from the beginning to the end of the sperm track (**D**). Track speed (VCL) is the average velocity measured over the actual point to point sperm track (**E**). Lateral amplitude (ALH) is the mean wdth of the head oscillation as the sperm swims (**F**). Beat frequency (BCF) is the frequency of sperm heads crossing the average sperm path in either direction (**G**). Straightness (STR) is a measure of the departure of the average sperm path from a straight line (ratio of VSL/VAP) (**H**). Linearity (LIN) is a measure of the departure of the actual sperm track from a straight line (ratio of VSL/VCL) (**I**). Elongation is the ratio of head width to head length (**J**). Values are given as the mean ± SD (n = 8-10). *p < 0.05 compared with AR-low.

Then, to compare motility of the sorted three groups of sperm, they were analysed by computer-assisted sperm analyser in multiple parameters. All the parameters of sperm motility were not different among the three groups (Fig. 4).

### Fertility and developmental abilities of acrosome-reacted sperm

**Figure 5:**
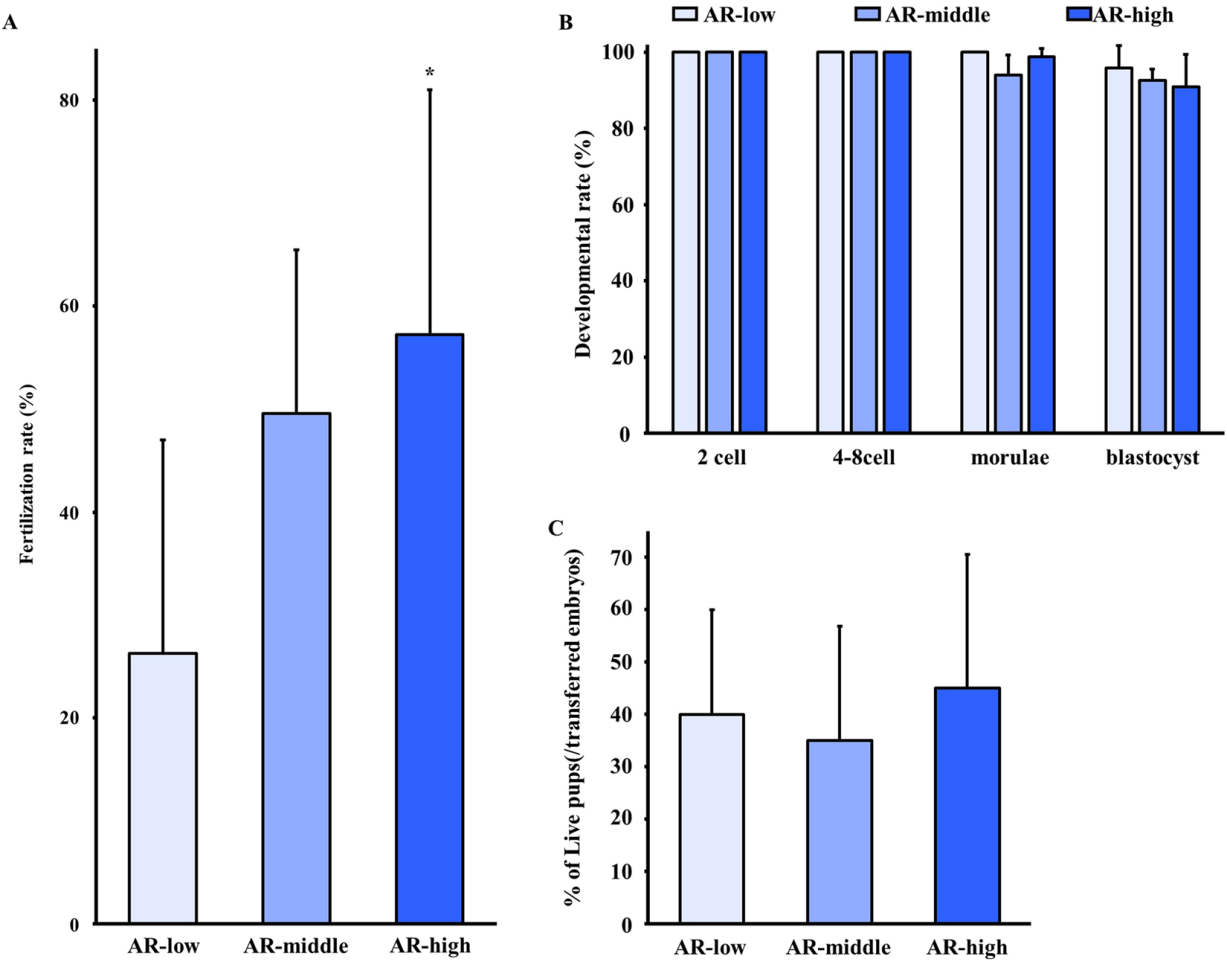
Effect of selection on sperm fertility and developmental ability. After selection, collected sperm suspensions were used as IVF medium. Oocytes were introduced into the IVF medium. Following IVF, the two-cell embryos were either cultured in KSOM/AA medium or transferred into the oviducts of pseudopregnant ICR females (10 embryos/oviduct). (**A**) The fertilisation rate was calculated as the number of two-cell embryos divided by the number of inseminated oocytes x 100. (**B**) The developmental rate was calculated as the number of embryos at several stages divided by the number of two-cell embryos x 100. (**C**) Two-cell embryos developed normally into live pups. The birth rate was calculated as the (number of live pups divided by the number of transferred two-cell embryos) x 100. Values are given as the mean ± SD (n = 3-5). *p < 0.05 compared with AR-low.

To study the effect of sperm sorting using a marker of AR on fertilisation ability, we conducted IVF using the AR-low, AR-middle and AR-high sperm. The fertilisation rate of the AR-high sperm was higher than was that of the AR-low sperm (Fig. 5A). The two-cell embryos developed normally into blastocysts and pups (Fig. 5B). When the embryos were transferred to the pseudopregnant females, we obtained normal pups with similar rate among three groups (Fig. 5C). These observations suggest that AR-based sperm sorting is effective to select fertilisation-prone sperm.

## Discussion

Sperm selection by flow cytometry is usually used for measuring DNA content^11–13^. Difference in the DNA content between X- and Y-chromosome sperm has been applied in the sex selection method used for rabbits, pigs, swine, cattle, sheep and horses^14–16^, combined with assisted reproductive technologies, such as IVF, artificial insemination or intracytoplasmic sperm injection to produce offsprings^17–19^. However, to the best of our knowledge, there have been no reports on sperm selection using flow cytometry and the use of the sorted sperm to produce embryos in mice using IVF.

In this study, we demonstrated that the microfluidics chip cell sorter is useful for sperm selection. The selected sperm showed *in vitro* fertility and full developmental ability. To the best of our knowledge, this may be the first report that sorted mouse sperm could produce healthy embryos and pups via IVF.

In addition, we demonstrated that sperm selection of AR-high sperm indicated higher fertilisation rate than that of AR-low sperm (Fig. 5). AR is a morphological change in sperm that occurs during sperm capacitation^20–23^ and is essential for fertilisation^24^. AR is induced by physiological and chemical stimulations such as interaction of ZP, progesterone or calcium ionophore^25–27^. However, the timing of AR is flexible and largely unknown *in vivo*. In mice sperm, it was clarified that most fertilising sperm initiate AR before contacting ZP and acrosome-reacted sperm can pass through ZP and fertilise eggs^28^. On the other hand, acrosome-intact and acrosome-reacted sperm are heterogeneously present with oocytes in IVF medium. We hypothesised that the presence of acrosome-intact sperm negatively affects to success of fertilisation. In this study, we first demonstrated that the sorted sperm from the AR-high group indicated higher fertilisation ability than those from the AR-low group in IVF. This finding implies that the selection of AR sperm is an important step to achieve fertilisation.

In this study, we demonstrated that FITC-labelled PNA sperm sorted using the microfluidic cell sorter had good motility, fertilisation and developmental abilities. FITC is the most widely used fluorescence probe conjugated with a small compound, protein or an antibody^29^. A study indicated that FITC does not impair the motility and fertilising functions of human sperm^30^. These results suggest that FITC and FITC-labelled compounds are useful to label sperm with normal functions. However, further investigations are needed to safely use the technique in industrial and medical applications.

Recently, Umehara reported a novel technique of sex selection based on Toll-like receptors 7/8 (TLR7/8)^31^. The TLR7/8 ligand suppressed the motility of X-chromosome-bearing sperm. The difference in motility between X- and Y-chromosome-bearing sperm after reagent treatment was applicable to produce pups of selected sex. Using the technique, 83% (XY, male) or 81% (XX, female) of pups were of the expected sex. Selection of TLR7/8-positive or TLR7/8-negative sperm by the microfluidic cell sorter may be a new application of sex sorting for IVF and embryo transfer and may improve the success rate of sex selection.

In conclusion, we showed that the microfluidics chip cell sorter is helpful in sorting and selecting mouse sperm. Application of the technology can improve the IVF technique by selecting good quality sperm. We consider that this sperm sorting technique represents an innovative approach to solving technical problems with reproductive technology used in various mammalian species.

## Methods

### Animals

C57BL/6J mice were purchased from CLEA Japan Inc. (Tokyo, Japan) and used as donors of sperm and oocytes. Female and male mice were 8–14 weeks old. Jcl:ICR mice (8–16 weeks old) were used as recipients of two-cell embryos. The mice were housed under a 12-h dark-light cycle (light from 07:00 to 19:00) at a constant temperature of 22 ± 1 °C, with free access to food and water. The Animal Care and Use Committee of Kumamoto University School of Medicine approved the protocols for animal experiments (ID: A2019-026), and all methods were performed in accordance with relevant guidelines and regulations.

### Reagents and media

BSA-free Toyoda Yokoyama Hosi medium, a modified Krebs–Ringer bicarbonate solution (fTYH) with 0.75 mM methyl-beta-cyclodextrin and 1.0 mg/mL polyvinyl alcohol (Sigma) (cTYH), was used as a medium for pre-incubation of sperm^32–34^. Calcium-enhanced human tubal fluid (mHTF) was used as the fertilisation medium^35–37^, and potassium simplex optimised medium (KSOM) was used for *in vitro* culture and embryo transfer^38^.

### Sperm sorting

Enumeration and sorting of sperm were performed using the microfluidics chip cell sorter (On-Chip Sort, On-chip Biotechnologies, Japan). The fluid channel was pre-washed with mHTF, and sperm samples were dissolved in mHTF so that the flow rate was approximately 400 events/s. The sperm were gated into scatter and collected in the collection reservoir. Collected sperm were fixed into 200 µL added mHTF and put it on the dish and covered with mineral oil. The dish was using as a fertilisation dish and the sperm was assessed the motility.

### Sperm selection based on AR

Sorting of sperm was conducted using the microfluidics chip cell sorter The fluid channel was pre-washed with mHTF, and sperm samples were dissolved in mHTF so that the flow rate was approximately 400 events/s. The sperm were gated into scatter or fluorescence intensity of FITC-labelled PNA (Figs. 3A,B) and collected in the collection reservoir. We classified them into three groups— AR-low, AR-middle and AR-high—by intensity of FITC (Fig. 3C–E). Collected sperm were fixed into 200 µL added mHTF and put it on the dish and covered with mineral oil. The dish was using as a fertilisation dish and the sperm was assessed the motility and the AR.

### Assessment of sperm motility

Sperm motility was assessed using a computer-assisted sperm analyser (IVOS Sperm Analyzer, Hamilton-Thorne Research Co. Ltd., USA)^39^. Sperm were incubated in cTYH for 60 min at 37 °C under 5% CO_2_ in air. Sperm suspensions were diluted 50 times in mHTF, incubated for 5 min at 37 °C and sorted using the microfluidics chip cell sorting system described previously. After sorting, sperm suspensions were placed in a disposable sperm analysis chamber (Hamilton-Thorne Research) and analysed using the IVOS system. We analysed the following sperm motility parameters: percentage of motile sperm (motile sperm moved more than 5-µm/s), percentage of motile sperm with progressive motility (motile sperm with progressive motility were denoted by a path velocity> 50 µm/s and a straightness ratio> 50%) and a marker of hyperactivation [lateral amplitude of head (ALH): this is the average value of the maximum swing width of the sperm head]. In addition, path velocity (VAP), progressive velocity (VSL), track speed (VCL), beat frequency (BCF), straightness (STR), Linearity (LIN) and Elongation were measured. During motility analysis, the sperm analysis chamber was warmed at 37 °C. To analyse the motile parameters, 200–1000 sperm were examined in each experiment. The experiments were independently from 7 to 10 times.

### Assessment of acrosome-reacted sperm

Acrosome-reacted sperm were evaluated using the peanut agglutinin FITC method^40^. Epididymal sperm were collected in 90 µl of cTYH, and the sperm suspensions were incubated for 60 min at 37 °C under 5% CO_2_ in air. Then, 10 µl of fTYH containing FITC-labelled PNA (10 µg/mL) was added to the drop of sperm suspension and incubated for 10 min. Sperm suspensions were diluted 50 times in mHTF and incubated for 5 min at 37 °C. Following incubation, sperm selection was conducted using the microfluidics chip cell sorting system and acrosome-reacted sperm (FITC-labelled PNA positive) were observed under fluorescence microscopy (Keyence Co. Ltd., Japan) to count the number of acrosome-intact sperm [AR(−)] (Fig. 3F,G) and acrosome-reacted sperm [AR (+),] (Fig. 3H,I). Percentage of acrosome-reacted sperm was calculated by the number of AR (+) sperm divided by the number of AR (+) sperm and number of AR (−) sperm.

### IVF

Female mice were superovulated via an intraperitoneal injection of 7.5 IU equine chorionic gonadotropin (ASKA Pharmaceutical Co. Ltd. Tokyo, Japan) and injected with 7.5 IU human chorionic gonadotropin (hCG, ASKA Pharmaceutical Co. Ltd., Tokyo, Japan) 48 h later. Then, 14–17 h after the hCG injection, the mice were euthanised by cervical dislocation. Their oviducts were collected and transferred to paraffin oil in a fertilisation dish that contained sorted sperm. Cumulus–oocyte complexes were collected from the oviducts into a drop of sorted sperm suspension (fertilisation drop) and covered with paraffin oil in the fertilisation dish. After 3 h, the oocytes were collected and washed three times in freshly prepared 100-µL drops of mHTF covered with paraffin oil. At 24 h after co-incubation, the fertilisation rate was calculated by the following equation: fertilisation rate (%) = the total number of two-cell embryos/the total number of inseminated oocytes × 100.

### Embryo transfer

To evaluate the developmental ability of two-cell embryos produced by IVF using sorted sperm, we performed embryo culture and transfer. After IVF, the two-cell embryos were divided into two groups, cultured to the blastocyst stage in a 100-µL drop of KSOM and transferred through the wall of the fallopian tube of pseudopregnant mice on the day in which a vaginal plug was found (10 embryos/oviduct)^41^. Then 19 days after embryo transfer, the number of offspring was recorded, and the birth rate was calculated by the following equation: birth rate (%) = the total number of offspring/the total number of transferred two-cell embryos × 100.

### Statistical analysis

Statistical analysis was performed using the statistical software Stat View-5.0 J (SAS Institute Inc., USA). The results are expressed as the mean ± SD. Differences between the means for each treatment were compared using analysis of variance following arcsine transformation of the percentages. When comparison of more than one pair of means was required, Tukey–Kramer’s test for pairwise comparisons was performed on the means, with p < 0.05 indicating significance.

## Supplementary information

Supplementary information1.

Supplementary information2.

## References

[1] Mahadevan, M & Baker, G Assessment and preparation of semen for in *vitro* fertilization in *clinical in vitro fertilization* (eds. Wood, C. & Trounson, A) 83–97 (*Springer-Verlag*, 1984).

[2] Bongso A (1989). Improved sperm concentration, motility, and fertilization rates following Ficoll treatment of sperm in a human *in vitro* fertilization program. Fertil. Steril..

[3] Gellert-Mortimer ST, Clarke GN, Baker HW, Hyne RV, Johnston WI (1988). Evaluation of Nycodenz and Percoll density gradients for the selection of motile human spermatozoa. Fertil. Steril..

[4] Hyne RV, Stojanoff A, Clarke GN, Lopata A, Johnston WI (1986). Pregnancy from *in vitro* fertilization of human eggs after separation of motile spermatozoa by density gradient centrifugation. Fertil. Steril..

[5] Takeda, K. & Jimma, F. *Maintenance free biosafety flow cytometer using disposable microfluidic chip (FISHMAN-R)*. Vol. 76B (2009).

[6] Watanabe M (2014). A novel flow cytometry-based cell capture platform for the detection, capture and molecular characterization of rare tumor cells in blood. J. Transl. Med..

[7] Takao M, Takeda K (2011). Enumeration, characterization, and collection of intact circulating tumor cells by cross contamination-free flow cytometry. Cytometry A.

[8] Watanabe M (2018). Isolation and molecular analysis of circulating tumor cells from lung cancer patients using a microfluidic chip type cell sorter. Cancer Sci.

[9] Kasuga Kie, Katoh Yasutake, Nagase Keisuke, Igarashi Kazuhiko (2017). Microproteomics with microfluidic-based cell sorting: Application to 1000 and 100 immune cells. PROTEOMICS.

[10] Takeda K, Fujimura Y, Jimma F (2011). Development of a new compact flow cytometer a using disposable micofluidic chip for contamination - free and biosafety measurement. Cytometry Research.

[11] Pinkel D (1979). Flow cytometry of mammalian sperm: progress in DNA and morphology measurement. J Histochem Cytochem.

[12] Van Dilla MA (1977). Measurement of mammalian sperm deoxyribonucleic acid by flow cytometry. Problems and approaches. J. Histochem. Cytochem..

[13] Evenson DP, Darzynkiewicz Z, Melamed MR (1980). Relation of mammalian sperm chromatin heterogeneity to fertility. Science.

[14] Seidel GE (1999). Insemination of heifers with sexed sperm. Theriogenology.

[15] Welch GR, Johnson LA (1999). Sex preselection: laboratory validation of the sperm sex ratio of flow sorted X- and Y-sperm by sort reanalysis for DNA. Theriogenology.

[16] Johnson LA (2000). Sexing mammalian sperm for production of offspring: the state-of-the-art. Anim. Reprod. Sci..

[17] Cran DG, Johnson LA, Miller NG, Cochrane D, Polge C (1993). Production of bovine calves following separation of X- and Y-chromosome bearing sperm and *in vitro* fertilisation. Vet Rec.

[18] Lindsey AC (2002). Hysteroscopic insemination of mares with low numbers of nonsorted or flow sorted spermatozoa. Equine Vet. J.

[19] DG C (1997). Production of lambs by low dose intrauterine insemination with flow cytometrically sorted and unsorted semen. Theriogenology.

[20] Ryuzo, Y. *Mammalian Fertilization*. Vol. 1 189–317 (Raven Press, 1994).

[21] E, E. & D, O. B. *Spermatozoon*. 1994 (Raven Press).

[22] Eisenbach M, Giojalas LC (2006). Sperm guidance in mammals - an unpaved road to the egg. Nat Rev Mol Cell Biol.

[23] Hirohashi N, Yanagimachi R (2018). Sperm acrosome reaction: its site and role in fertilization. Biol. Reprod..

[24] M. DJ (1991). A Test of the human sperm acrosome reaction following ionophore challenge. J. Androl..

[25] Bleil JD, Wassarman PM (1983). Sperm-egg interactions in the mouse: sequence of events and induction of the acrosome reaction by a zona pellucida glycoprotein. Dev. Biol..

[26] Osman RA, Andria ML, Jones AD, Meizel S (1989). Steroid induced exocytosis: the human sperm acrosome reaction. Biochem. Biophys. Res. Commun..

[27] Tateno H (2013). Ca2+ ionophore A23187 can make mouse spermatozoa capable of fertilizing *in vitro* without activation of cAMP-dependent phosphorylation pathways. Proc. Natl. Acad. Sci. U S A.

[28] Inoue N, Satouh Y, Ikawa M, Okabe M, Yanagimachi R (2011). Acrosome-reacted mouse spermatozoa recovered from the perivitelline space can fertilize other eggs. Proc. Natl. Acad. Sci. U S A.

[29] Sabnis, R. W. Fluoroscein-5-isothiocyanate (FITC), In Handbook of Fluorescent Dyes and Probes, R.W. Sabnis (Ed.), 10.1002/9781119007104.ch80 (2015).

[30] BLAZAK W. F., OVERSTREET J. W., KATZ D. F., HANSON F. W. (1982). A CompetitiveIn VitroAssay of Human Sperm Fertilizing Ability Utilizing Contrasting Fluorescent Sperm Markers. Journal of Andrology.

[31] Umehara T, Tsujita N, Shimada M (2019). Activation of Toll-like receptor 7/8 encoded by the X chromosome alters sperm motility and provides a novel simple technology for sexing sperm. PLoS Biol..

[32] Yutaka T, Minesuke Y, Tosiro H (1971). Studies on the fertilization of mouse eggs *in vitro* I. *in vitro* fertilization of eggs by fresh epididymal sperm. The Japanese Journal of Animal Reproduction.

[33] Toyoda, Y. & Yokoyama, M. *The early history of the tyh medium for in vitro fertilization of mouse ova*. Vol. 33 (2016).

[34] Takeo T (2008). Methyl-beta-cyclodextrin improves fertilizing ability of C57BL/6 mouse sperm after freezing and thawing by facilitating cholesterol efflux from the cells. Biol. Reprod..

[35] Takeo T, Nakagata N (2010). Combination medium of cryoprotective agents containing L-glutamine and methyl-{beta}-cyclodextrin in a preincubation medium yields a high fertilization rate for cryopreserved C57BL/6J mouse sperm. Lab. Anim..

[36] Quinn P, Kerin JF, Warnes GM (1985). Improved pregnancy rate in human *in vitro* fertilization with the use of a medium based on the composition of human tubal fluid. Fertil. Steril..

[37] Kito S (2004). Improved *in vitro* fertilization and development by use of modified human tubal fluid and applicability of pronucleate embryos for cryopreservation by rapid freezing in inbred mice. Comp. Med.

[38] Lawitts JA, Biggers JD (1993). Culture of preimplantation embryos. Methods Enzymol.

[39] Itach SB, Finklestein M, Etkovitz N, Breitbart H (2012). Hyper-activated motility in sperm capacitation is mediated by phospholipase D-dependent actin polymerization. Dev. Biol..

[40] Kallajoki M, Virtanen I, Suominen J (1986). The fate of acrosomal staining during the acrosome reaction of human spermatozoa as revealed by a monoclonal antibody and PNA-lectin. Int. J. Androl..

[41] Nakagata N (1992). [Embryo transfer through the wall of the fallopian tube in mice]. Jikken Dobutsu.

